# Protein Biofortification in Lentils (*Lens culinaris* Medik.) Toward Human Health

**DOI:** 10.3389/fpls.2022.869713

**Published:** 2022-04-05

**Authors:** Sonia Salaria, Jon Lucas Boatwright, Pushparajah Thavarajah, Shiv Kumar, Dil Thavarajah

**Affiliations:** ^1^Plant and Environmental Sciences, Clemson University, Clemson, SC, United States; ^2^Biodiversity and Crop Improvement Program, International Centre for Agricultural Research in the Dry Areas (ICARDA), Rabat-Institute, Rabat, Morocco

**Keywords:** Lentil (*Lens culinaris L*.), protein, biofortification, amino acids, protein quality, food secuity

## Abstract

Lentil (*Lens culinaris* Medik.) is a nutritionally dense crop with significant quantities of protein, low-digestible carbohydrates, minerals, and vitamins. The amino acid composition of lentil protein can impact human health by maintaining amino acid balance for physiological functions and preventing protein-energy malnutrition and non-communicable diseases (NCDs). Thus, enhancing lentil protein quality through genetic biofortification, i.e., conventional plant breeding and molecular technologies, is vital for the nutritional improvement of lentil crops across the globe. This review highlights variation in protein concentration and quality across *Lens* species, genetic mechanisms controlling amino acid synthesis in plants, functions of amino acids, and the effect of antinutrients on the absorption of amino acids into the human body. Successful breeding strategies in lentils and other pulses are reviewed to demonstrate robust breeding approaches for protein biofortification. Future lentil breeding approaches will include rapid germplasm selection, phenotypic evaluation, genome-wide association studies, genetic engineering, and genome editing to select sequences that improve protein concentration and quality.

## Introduction

Nutritional imbalances and deficiencies cause several malnutritional and non-communicable diseases (NCDs) in humans. A poor diet that lacks macro- and micronutrients, such as proteins, low-digestible carbohydrates (LDCs), fats, vitamins, and minerals, results in protein and micronutrient malnutrition. Low-digestible carbohydrates (LDs) are, also known as prebiotic carbohydrates, defined as ‘a substrate that is selectively utilized by host microorganisms conferring a health benefit’ (Gibson et al., 2017). These dietary prebiotic carbohydrates pass undigested through the upper digestive tract and are fermented by microorganisms in the colon for increased gut health. The most common human health impacts of malnutrition are stunting, intestinal health issues impairing digestion, obesity, overweight, and an increased risk of diet-related NCDs (Branca et al., 2019). Major NCDs related to poor dietary intake that threatens human life include cardiovascular diseases, cancer, chronic respiratory diseases, and diabetes (World Health Organization, 2019). Notably, a protein-deficient diet leading to protein malnutrition has alarming consequences that affect infants, young children, and females across the globe (Semba, 2016). However, a protein-rich legume-based diet is a viable, sustainable, and healthy option to prevent malnutrition in developing countries. Though animal proteins are extensively utilized in human diets, plant-based proteins have grown in popularity. Their demand has increased globally due to nutritional value, low carbon input, and environmental concerns (Asif et al., 2013).

Staple foods rich in macro- and micronutrients can alleviate the risk of malnutrition. Plant-based diets comprised mainly of cereals and legume staples are popular worldwide. Legume crops, including lentils (*Lens culinaris* Medikk.), have a protein concentration (20–30%) higher than cereals (10–12%) and thus have the potential to combat protein malnutrition and serve as gluten- and allergen-free protein sources. Lentil is highly nutritious, affordable and has a shorter cooking time than other pulse crops, and features high protein concentrations, low-digestible carbohydrates, minerals, vitamins, and low concentrations of phytic acid (Thavarajah et al., 2009; Kumar et al., 2015). Lentil is not a source of cholesterol, and its low-fat content makes it easier to digest than other pulse crops. Lentil proteins include both essential and non-essential amino acids but are notably low in the sulfur-containing amino acids methionine (Met) and cysteine (Cys; Khazaei et al., 2019). Biofortification is a possible approach to improve the unbalanced composition of amino acids in lentils through appropriate conventional breeding strategies and genomic selection. With increasing global protein demand, protein biofortification would justify lentils as a ‘*nutritional booster*’ to increase global nutritional security and combat malnutrition and NCDs.

Lentil proteins are stored in the cotyledonary cells in membranous protein bodies called ‘storage proteins’ (Duranti and Gius, 1997). These seed proteins supply carbon (C), nitrogen (N), and sulfur (S) and compose 80% of the total protein available for germination, subsequent plant growth, and disease resistance (Khazaei et al., 2019). Storage proteins also play a defensive role against bruchids, insects of the family Bruchidae, in cowpea (*Vigna unguiculata*; Sales et al., 2000). These proteins are classified into four types: globulins (salt soluble), albumins (water-soluble), prolamins (ethanol soluble), and glutelins (acid-soluble; Osborne, 1924). Like other pulse crops, lentils are rich in globulins and albumins, whereas prolamins and glutelins are more prominent in cereals (Osborne, 1924). Globulins were the first type of storage protein reported in lentils (Osborne and Campbell, 1898) and are the principal proteins in lentils, making up ~44–70% of all storage proteins. Two subclasses of globulins, i.e., 11 s type (legumin) and 7 s type (vicilin/convicilin), were also defined (Danielson, 1950). Albumins comprise 26–61% of lentil proteins, and prolamins and glutelins only make up a small fraction (Saint-Clair, 1972; Sulieman et al., 2008).

Storage protein quantities demonstrate high variability due to the quantitative nature of the genes regulating protein synthesis in the seeds (Kumar et al., 2020). Higher genotype × environmental interactions, indicated by the moderate broad sense heritability (31.31%), is another reason for the high variation in the storage protein concentration in lentil seeds (Gautam et al., 2018). Lentil seed proteins, excluding storage proteins, also have metabolic functions. These metabolic proteins regulate numerous physiological processes in the plant, including enzymatic activity and structural and physiological functions (Scippa et al., 2010). Ultimately, lentil seed protein composition contributes to human health by providing essential amino acids necessary for metabolic processes and nutritional balance in the human body. Optimizing the plant breeding process and location sourcing may help develop better protein-enriched lentil cultivars for global plant-based protein demand. The objectives of this paper are to review the protein concentration and quality variations within the genus *Lens*, pathways and genes regulating the synthesis of amino acids, functions of amino acids for human health, and breeding strategies related to lentil protein biofortification.

## Lentil Biofortification

Lentil is an annual diploid (2n = 2x = 14) cool-season food legume that originated in the Middle East (Cubero, 1981). The genus *Lens* comprises *L. culinaris*, *L. ervoides*, *L. nigricans*, and *L. lamoletti*. *L. culinaris* is further divided into four taxa: *L. culinaris* ssp. *culinaris*, *L. culinaris* ssp. *orientalis*, *L. culinaris* ssp. *tomentosus*, and *L. culinaris* ssp. *odemensis* (Ferguson et al., 2000). Lens genus has been classified as primary, secondary, tertiary, and quaternary genetic pools according to the phylogeny using the Genotyping-by-sequencing (GBS). The primary gene pool contains *L. culinaris*, *L. orientalis*, and *L. tomentosus*, whereas *L. odemensis* and *L. lamoletti* are in the secondary gene pool. However, each tertiary and quaternary gene pools contain single species, *L. ervoides* and *L. nigricans*, respectively (Wong et al., 2015). Of these, only *L. culinaris* ssp. *culinaris* is domesticated and cultivated worldwide, representing crops over a 5.01 M ha area with an annual production of 6.54 M tonnes. Canada is a leading producer, contributing about 44% of the world’s lentils; other major lentil-producing countries are India, the United States of America (United States), Turkey, Australia, Nepal, and Bangladesh (FAOSTAT, 2021).

Lentils are a staple food that is easily digested compared to other legumes. The biofortification of lentils could significantly fight hidden hunger and nutritional disorders. Hidden hunger is also known as micronutrient deficiency despite sufficient calorie intake (Lowe, 2021). Several breeding programs have been established worldwide that seek to biofortify lentils with protein, prebiotic carbohydrates, micronutrients, vitamins, etc. (Kumar et al., 2016a). Many lentil accessions have been screened for amino acid concentration (Iqbal et al., 2006), protein (Bhatty and Slinkard, 1979), starch (Zia-Ul-Haq et al., 2011), fatty acids (Grusak, 2009), macro- and micronutrients (Kumar et al., 2016a; Podder et al., 2020; Rasheed et al., 2020), folates (Sen Gupta et al., 2013), and antinutritional factors (Thavarajah et al., 2009, 2011). Marker-assisted breeding has also demonstrated the potential for identifying genes/quantitative trait loci (QTL) for iron (Fe) uptake (Kumar et al., 2015; Aldemir et al., 2017), Fe and Zinc (Zn) concentration (Kumar et al., 2014), and selenium (Se) concentration (Ates et al., 2016). Furthermore, the HarvestPlus Challenge program, established in 2004, was a landmark effort that increased lentil biofortification efforts worldwide. They released several lentil cultivars to economically underprivileged global regions in Asia and Africa (Kumar et al., 2016a). Notably, numerous high Fe and Zn cultivars have been released, including Barimasur-4, -5, -6, -7, -8 and -9 in Bangladesh; Khajuraho-1, -2 and -3, Sital, Shekhar, Sisir, and Simal in Nepal; L 4704, IPL 220, Pusa Agaiti and Pusa Vaibhav in India; Idlib-2 and -3 in Syria/Lebanon; and Alemeya in Ethiopia. Smallholder farmers regularly use these biofortified lentils in Africa and Southeast Asia (Harvest Plus, 2014).

Various researchers have reported protein concentrations in current lentil cultivars in the range of 20–30% (Table 1). In a study (Bhatty, 1986), similar protein concentrations in wild and cultivated lentils, indicating homogeneity for protein concentration in the genus *Lens*, were identified. However, a recent study (Kumar et al., 2016b) efficiently distinguished wild species from cultivated lentils for protein concentration. In this study, *L. orientalis*, an immediate progenitor of cultivated lentils, expressed the highest average protein (24.15%) among all the wild species, followed by *L. ervoides* (22.99%). Other wild species, *L. odemensis*, and *L. nigricans* showed slightly higher average protein content (19.7 and 19.53%, respectively) than *L. culinaris*. A similar protein level was seen in *L. tomentosus* (18.75%) and cultivated lentils (18.7%). Extensive variation was observed for protein content within *L. orientalis* and *L. ervoides*, ranging from 18.3 to 27.75% and 18.9 to 32.7%, respectively. ILWL-47, an *L. ervoides* accession, had an exceptionally high protein content of about 32.7% and is, therefore, a potential candidate for protein quality improvement in lentil breeding programs (Kumar et al., 2016b). Protein subunit fraction profiling has indicated variable levels of the albumin protein fraction (APF) and globulin protein fraction (GPF) among *Lens* species, with the wild species having higher APF and GPF concentrations than the cultivated species (Bhatty, 1982). Among the evaluated wild species, *L. orientalis* and *L. ervoides* contained higher APF and GPF levels than *L. nigricans* (Bhatty, 1982).

**Table 1:** Genetic variation for protein concentration in cultivated lentils (*L. culinaris*).

Protein concentration (% of dry matter)	Total accessions used	Reference
24.6–30.0	23	Heuzé et al., 2021
10.5–27.1	45	Kumar et al., 2016b
21.8–27.1	14	Zaccardelli et al., 2012
25.3–29.3	35	Alghamdi et al., 2014
23.8–29.3	22	Tahir et al., 2011
24.3–30.2	4	Wang and Daun, 2006
23.9–26.3	58	Stoddard et al., 1993
25.5–28.9	24	Erskine et al., 1985

The proportion of amino acids in lentil proteins varies across genotypes in the cultivated gene pool (Table 2). Met and tryptophan (Trp) represent a minor fraction among all amino acids and are thus termed limiting amino acids. Comparing lentil protein with cereal proteins indicates the good nutritional complementation between Met and lysine (Lys), but to some extent, for Trp and threonine (Thr) because cereals are rich in both Met and Trp (Bhatty, 1986). Generally, all essential amino acids except Lys are deficient in lentils, but a moderate to the high proportion of non-essential amino acids are present (Khazaei et al., 2019). Lentil proteins are also lacking in other S-containing amino acids such as Cys. The albumin fraction of lentils contains more essential amino acids than the globulin fraction (Bhatty, 1982). Recent studies also indicate that amino acids vary among distinct species of the genus *Lens*, with a spectrum of variation seen for amino acid content among *L. culinaris*, *L. orientalis*, *L. ervoides*, *L. nigricans*, and *L. odemensis*. Phenylalanine (Phe), Met, valine (Val), leucine (Leu), and isoleucine (Ile) concentrations are significantly higher in wild species than cultivated lentils (Table 3; Rozan et al., 2001). Similarly, the non-essential amino acid content is also higher in wild species than in *L. culinaris*. Such evidence signifies wild species are a potential source of candidate genes that can be harnessed to improve protein quality in cultivated lentils.

**Table 2:** Amino acid profile of cultivated lentil genotypes (Sayeed and Njaa, 1985; Shekib et al., 1986; Kahraman, 2018).

Amino acids	Concentration (g/100 g of protein)
Ala	3.31–8.35
Arg	4.64–13.80
Asp	6.36–13.20
Cys	0.60–1.62
Glu	6.12–17.10
Gly	4.40–10.40
His	1.21–9.15
Ile	2.20–5.00
Leu	5.21–7.72
Lys	5.81–9.59
Met	0.90–2.23
Phe	3.85–7.55
Pro	3.50–5.22
Ser	4.90–6.34
Thr	1.04–4.60
Trp	0.57–1.37
Tyr	2.71–7.15
Val	4.10–5.01

*Ala, alanine; Arg, arginine; Asp, aspartate/aspartic acid; Cys, cysteine; Glu, glutamate/glutamic acid; Gly, glycine; His, histidine; Ile, isoleucine; Leu, leucine; Lys, lysine; Met, methionine; Phe, phenylalanine; Pro, proline; Ser, serine; Thr, threonine; Trp, tryptophan; Tyr, tyrosine; Val, valine*.

**Table 3:** Amino acid concentrations among different *Lens* species (Rozan et al., 2001).

Amino acids	L. culinaris	L. orientalis	L. ervoides	L. nigricans	L. odemensis
		mg amino acids/g of dry seed weight			
Ala	20.42	39.81	16.01	22.47	21.32
Arg	10.61	14.04	12.05	7.48	9.10
Asp	10.96	26.10	17.42	7.68	11.17
Cys	0.40	0.39	0.53	0.47	0.44
Glu	26.55	42.27	32.62	19.95	24.22
Gly	9.77	12.66	11.48	7.89	10.22
His	8.74	3.95	9.75	4.94	6.84
Ile	6.26	9.58	8.59	7.76	5.06
Leu	10.64	15.86	14.07	11.74	8.09
Lys	4.54	12.64	9.48	6.14	5.69
Met	1.49	1.63	1.74	1.22	1.18
Phe	6.70	10.64	9.37	9.46	5.55
Pro	11.11	11.36	11.54	10.52	8.88
Ser	11.38	15.60	14.10	8.70	11.20
Thr	5.57	7.57	6.31	4.56	5.62
Trp	NA	NA	NA	NA	NA
Tyr	6.34	7.53	6.65	6.35	5.05
Val	8.54	11.64	9.60	8.64	7.24

*Ala, alanine; Arg, arginine; Asp, aspartate/aspartic acid; Cys, cysteine; Glu, glutamate/glutamic acid; Gly, glycine; His, histidine; Ile, isoleucine; Leu, leucine; Lys, lysine; Met, methionine; Phe, phenylalanine; Pro, proline; Ser, serine; Thr, threonine; Trp, tryptophan; Tyr, tyrosine; Val, valine*.

## Genetic Control for Amino Acid Biosynthesis in Plants

The genetic mechanisms controlling seed protein concentration have similar regulation and pathways in different plants, including pulse crops. In pulse crops, genetic control of seed protein content has not been widely studied except in chickpea (*Cicer arietinum*), soybean (*Glycine max*), and pea (*Pisum sativum*). However, genetic control of seed protein content has been studied extensively in cereals (Mann et al., 2009; Olsen and Phillips, 2009; Chen et al., 2018; Borisjuk et al., 2019) and the model plant *Arabidopsis thaliana* (Jasinski et al., 2016). In chickpea, seven candidate genes that regulate seed protein concentration were identified using a genome-wide association study of 336 *desi* and *Kabuli* accessions (Upadhyaya et al., 2016). In soybean, three QTL (qPro10a, qPro13a, and qPro17b) for protein were identified in a recombinant inbred line (RIL) population (Zhonghuang 24 × Huaxia 3) on chromosomes 10, 13, and 17, respectively (Liu et al., 2017).

Several genes regulating the seed protein concentration in soybean were found on chromosomes 15 and 20 (Patil et al., 2017). Another gene, *BIG SEEDS1* (*BS1*), controlling seed size, weight, and composition of amino acids in the protein, has been characterized in *Medicago trunculata* and soybean (Ge et al., 2016). Groups of highly coordinated genes (HCGs) controlling the aspartate family (Met, Ile, Lys, Thr, and Gly) and branched aromatic amino acid formation were also identified in *A. thaliana* (Less and Galili, 2009). These two HCGs have several genes controlling the formation of amino acids. The first group related to the aspartate family contained catabolic genes for *THA1* (Thr to Gly metabolism), *BCAT2* (Ile metabolism), *MGL* (Met catabolism), and *LKR/SDH* (Lys metabolism). However, the second group exclusively regulated Met metabolism and was termed the ‘Met metabolism group.’ It contained the genes *AK/HSDH1* (encoding aspartate kinase enzyme for the formation of aspartate-4-semialdehyde, the first substrate for amino acid synthesis), *CGS1* (Met synthesis), *DAPD* (Lys synthesis), *SAMS3* (Met catabolism), *BCAT3* (Ile metabolism), and *BCAT4*, *MAM1*, and *MAML* (Met catabolism). One of the two groups related to branched aromatic acids contained ten genes (*ASA1*, *ASB*, *TSA2*, *TSB1/2*, *IGPS* for Trp synthesis, *CYP79B2* for Trp catabolism, *PD* for Phe synthesis, *PAL1* and *PAL2* for Phe catabolism, and *TAT3* for tyrosine (Tyr) catabolism). In contrast, two genes (*PAL3* and *IGPS*) were reported in the second group (Less and Galili, 2009).

**Figure 1:**
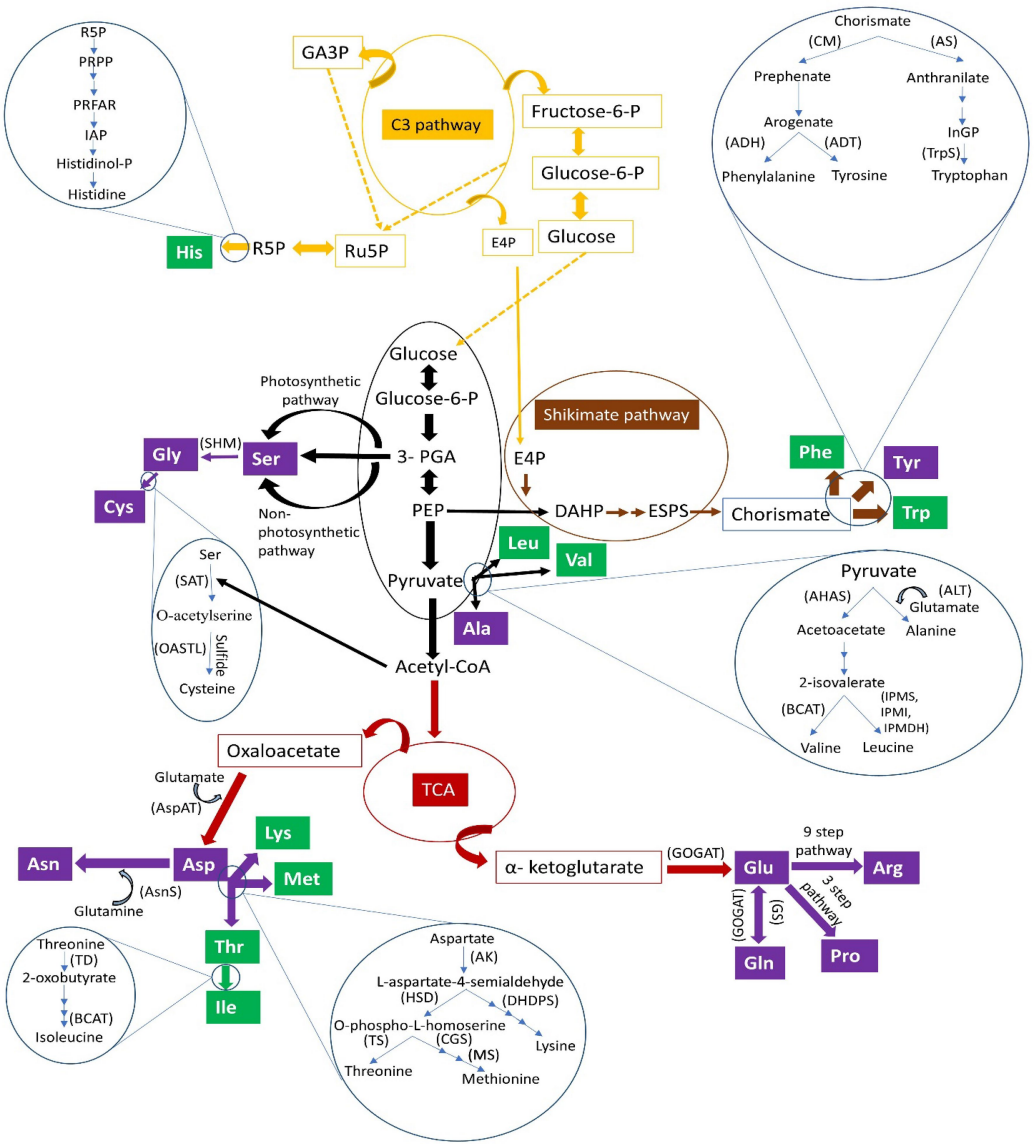
Pathways synthesizing various essential (green boxes) and non-essential (purple boxes) amino acids. Amino acids: Ala, alanine; Arg, arginine; Asn, asparagine; Asp, aspartate/aspartic acid; Cys, cysteine; Gln, glutamine; Glu, glutamate/glutamic acid; Gly, glycine; His, histidine; Ile, isoleucine; Leu, leucine; Lys, lysine; Met, methionine; Phe, phenylalanine; Pro, proline; Ser, serine; Thr, threonine; Trp, tryptophan; Tyr, tyrosine; Val, valine). Substrates/precursors: acetyl-CoA, acetyl-coenzyme A; DAHP, 3-deoxy-D-arabinoheptulosonate-7-phosphate; ESPS, 5-enolpyruvylshikimate-3-phosphate; E4P, erythrose 4-phosphate; fructose-6-P, fructose-6-phosphate; GA3P, glyceraldehyde 3-phosphate; glucose-6-P, glucose-6-phosphate; histidinol-P, histidinol phosphate; IAP, imidazole acetol-phosphate; PEP, phosphoenol pyruvate; 3-PGA, 3-phosphoglyceric acid; PRFAR, (*N´*-[(5′-phosphoribulosyl)formimino]-5-aminoimidazole-4-carboxamide) ribonucleotide); PRPP, phosphoribosyl diphosphate; R5P, ribose 5-phosphate; Ru5P, ribulose 5-phosphate. Enzymes indicated in parentheses: ADH, arogenate dehydrogenase; ADT, arogenate dehydratase; AHAS, acetohydroxyacid synthase; AK, aspartate kinase; ALT, alanine transferase; AS, anthranilate synthase; AsnS, asparagine synthetase; AspAT, aspartate aminotransferase; BCAT, branched-chain amino acid aminotransferase; CGS, cystathionine gamma synthase; CM, chorismate mutase; DHDPS, dihydrodipicolinate synthase; GOGAT, glutamate synthase; GS, glutamine synthetase; HSD, homoserine dehydrogenase; IPMS, isopropylmalate synthase; MS, methionine synthase; OASTL, O-acetylserine(thiol)lyase; SAT, serine acetyltransferase; SHM, serine hydroxymethyltransferase; TD, threonine deaminase; TrpS, tryptophan synthase; TS, threonine synthase.

The genes regulating the synthesis of enzymes that mediate the formation of amino acids and their precursors have been extensively studied in plants (Table 4; Figure 1). In *A. thaliana*, glutamate is formed from precursor 2-oxoglutarate by enzymatic aminotransferases, a process that is regulated by 44 putative genes (Liepman and Olsen, 2004). Glutamate synthase production, which converts glutamine (Gln) to glutamate, is controlled by either one or two genes in the chloroplast and mitochondria (Gaufichon et al., 2016). Similarly, six genes encode Gln synthase, which converts glutamate to Gln, in *A. thaliana* (Forde and Lea, 2007). Glutamate is a precursor that synthesizes arginine (Arg) and proline (Pro) using 20 enzymes encoded by about 30 genes in *A. thaliana* (Majumdar et al., 2016). Glutamine with aspartate also forms asparagine (Asn) in plants by the transamination action of the Asn synthetase (AS) enzyme encoded by the *asnB* gene in eukaryotes (Gaufichon et al., 2010) and the *ASN* gene family (*ASN1*, *ASN2*, and *ASN3*) in *Arabidopsis* (Table 4; Arabidopsis Genome Initiative, 2000). A histidine (His) synthesis pathway revealed eight genes (*ATP-PRT*, *PRATP/CH*, *ProFAR-I*, *IGPS*, *IGPD*, *HPA*, *HPP*, and *HDH*) forming eight enzymes in *A. thaliana* (Rees et al., 2009). Two branched-chain amino acids, Val and Leu, form with the acetohydroxyacid synthase (AHAS) enzyme acting on pyruvate producing acetolactate. This enzyme forms the third branched-chain amino acid, Ile, by serving on a substrate formed from Thr in the pathway for 2-ketobutyrate converting Thr to Ile. A single gene encodes the AHAS enzyme in *Arabidopsis* (Singh and Shaner, 1995).

**Table 4:** Genes responsible for amino acid synthesis.

Amino acid	Key precursors	Key enzymes	Genes in *Arabidopsis*	References
Glutamate	2-oxoglutarate; Glutamine	Amino transferases; glutamate synthase (GOGAT): two forms- ferredoxin (Fd) and NADH	44 putative genes Fd form: *GLU1*, *GLU2* NADH form: *GLT*	Liepman and Olsen, 2004; Forde and Lea, 2007
Glutamine	Glutamate	Glutamine synthase: two forms—plastidic (GS1) and cytoplasmic (GS2)	GS1 form: one gene; GS2 form: five genes	Forde and Lea, 2007; Gaufichon et al., 2016
Asparagine	Glutamine and Aspartate	Asparagine synthase	asnB gene; ASN gene family (*ASN1*, *ASN2*, *ASN3*)	Arabidopsis Genome Initiative, 2000; Gaufichon et al., 2010
Histidine	Ribose-5-phosphate	Eight enzymes	*PRATP/CH*, *ProFAR-I*, *IGPS*, *HPP*, *HDH*- single copy genes; *ATP-PRT*, *IGPD*, *HPA*-duplicated genes	Rees et al., 2009; Ingle, 2011
Leucine	Pyruvate 2-oxoisovalerate	Acetohydroxyacid synthase (AHAS) enzyme Isopropylmalate synthase (IPMS), isopropylmalate isomerase (IPMI), and isopropylmalate dehydrogenase (IPMDH)	*AHAS* gene IPMS: *IPMS1*, *IPMS2*, *IPMI LSU1 IPMI SSU1 IPMI SSU2*, *IPMI SSU3*, *IPMDH* gene	Singh and Shaner, 1995; Calder, 1995; Xing and Last, 2017; Knill et al., 2009
Valine	Pyruvate 2-oxoisovalerate	Acetohydroxyacid synthase (AHAS) enzyme Amino transferase	*AHAS* gene Single gene	Singh and Shaner, 1995; Calder, 1995; Xing and Last, 2017; Knill et al., 2009
Isoleucine	2-ketobutyrate	Acetohydroxyacid synthase (AHAS) enzyme	*AHAS* gene	Singh and Shaner, 1995
Alanine	Pyruvate and glutamate	Alanine aminotransferases	Eight genes	Parthasarathy et al., 2019
Phenylalanine Tyrosine	Chorismate Prephenate Arogenate	Chorismate mutase (CM), Prephenate aminotransferase Phenylalanine synthesis: Arogenate dehydratase Tyrosine synthesis: Arogenate dehydrogenase	CM: *AtCM1*, *AtCM2*, *AtCM3*, *AtPPA-AT* gene Six genes (*ADT1*, *ADT2*, *ADT3*, *ADT4*, *ADT5*, *ADT6*) Two genes (*TyrA1*, *TyrA2*)	Tzin and Galili, 2010; Dudareva et al., 2011
Tryptophan	Chorismate Anthranillite	Anthranilate synthase (AS) Anthranilate phosphoribosyltransferase (PAT1), indole-3-glycerol phosphate synthase (IGPS), tryptophan synthase alpha (TS a), phosphoribosylanthranilate isomerase (PAI), and tryptophan synthase beta (TS b)	Three genes (*ASa1*, *ASa2*, *ASb1*) and seven putative genes (two *Asa* and five *ASb* genes) *PAT1*, *IGPS*, *TSa*, three genes (*PAI1*, *PAI2*, *PAI3*) and two genes (*TSb1 and TSb2*)	Tzin and Galili, 2010; Parthasarathy et al., 2018
Aspartate	Oxaloacetate and glutamate	Aspartate aminotransferase (AspAT)	Five genes: *AspAT1*, *AspAT2*, *AspAT3*, *AspAT4*, *AspAT4*, *AspAT5*	Han et al., 2021
Methionine, Threonine, Isoleucine Lysine	Aspartate L-aspartate-4-semialdehyde	Aspartate kinase (AK) Methionine, threonine and isoleucine synthesis: homoserine dehydrogenase (HSD) Lysine synthesis: dihydrodipicolinate synthase (DHDPS)	Five genes Two genes Two genes	Vauterin and Jacobs, 1994; Vauterin et al., 1999; Craciun et al., 2000; Sarrobert et al., 2000; Galili, 2011

The enzyme chorismate mutase (CM) is encoded by three genes (*AtCM1*, *AtCM2*, and *AtCM3*) and is a precursor for chorismate to form prephenate for Phe and Tyr biosynthesis in plants (Figure 1). The formation of Trp from chorismate is regulated by three genes (*ASa1*, *ASa2*, and *ASb1*) and seven putative genes (two *Asa* and five *ASb* genes) encoding anthranilate synthase (AS) enzyme-producing anthranilate (Table 4). This anthranilate generates Trp using five enzymes (PAT1, PAI, IGPS, TS a, and TS b) encoded by eight genes in plants (Tzin and Galili, 2010; Parthasarathy et al., 2018). Aspartate regulates the formation of four essential amino acids, Ile, Lys, Met, and Thr, also termed aspartate-derived amino acids. Five genes encode aspartate formation enzymes in *A. thaliana* (Han et al., 2021). In C_3_ plants, including lentils, two pathways are identified for serine (Ser) formation, namely photorespiratory and non-photorespiratory pathways in photosynthetic and non-photosynthetic tissues, respectively (Figure 1). The Ser produced in different pathways is converted into glycine (Gly) in non-photosynthetic tissues in the presence of the Ser hydroxymethyltransferase (SHM) enzyme. Ser also synthesizes Cys by following a two-step pathway in plants regulated by Ser acetyltransferase (SAT) and O-acetylserine (thiol)lyase (OASTL) enzymes encoded by five and nine genes, respectively (Howarth et al., 1997; Wirtz et al., 2004).

## Amino Acids Impact Human Health

Amino acids are the foundational units of proteins. Structural conformations have unique chemical properties due to basic (amide) and acidic (carboxylic) chemical groups. Based on the human nutritional requirements, amino acids have been classified in several ways—essential or non-essential. Essential amino acids are indispensable because the human body cannot synthesize them; hence, appropriate concentrations in the diet are necessary (Table 5). Non-essential amino acids, synthesized in the human body, are also called dispensable amino acids (Reeds, 2000). However, some non-essential amino acids are considered conditionally non-essential because their abundance in the human body declines in times of stress or sickness. External sources are required to maintain necessary quantities (Fürst and Young, 2000).

**Table 5:** Classification of amino acids based on human nutritional requirements.

Class of amino acid	Amino acids	Abbreviations
Essential		
	Histidine	His
	Isoleucine	Ile
	Leucine	Leu
	Lysine	Lys
	Methionine	Met
	Phenylalanine	Phe
	Threonine	Thr
	Tryptophan	Trp
	Valine	Val
Conditionally essential		
	Arginine	Arg
	Cysteine	Cys
	Glycine	Gly
	Glutamine	Gln
	Proline	Pro
	Tyrosine	Tyr
Non-essential		
	Alanine	Ala
	Asparagine	Asn
	Aspartate/aspartic acid	Asp
	Glutamate/glutamic acid	Glu
	Serine	Ser

The role of amino acids (individually or in combination) was first studied in rats to evaluate the necessity of Lys and Trp in food sources containing gliadin proteins. This initial study documented the adverse effects of amino acid deficiency on rats (Osborne and Mendel, 1914). Based on preliminary classical studies using model organisms (Ackroyd and Hopkins, 1916; Rose and Cox, 1924), an analogy of amino acid functions and dietary requirements in humans was first established by Rose and co-workers in 1947 (Rose et al., 1947). This study played a significant role in recognizing and classifying essential and non-essential amino acids based on their impacts on human health. Amino acids perform several crucial functions in the human body, either directly or indirectly. Amino acids have a specific role in gene expression (Oommen et al., 2005), signaling pathways for activation of immune systems (Kim et al., 2007), have nutraceutical effects for improving health status by regulating metabolic activities (Duranti, 2006), and can be used to treat genetic disorders (van Vliet et al., 2014).

Amino acids govern the epigenetic regulation of gene expression through DNA modifications. DNA modifications such as methylation and acetylation occur due to the binding of DNA to C groups (methyl, acetyl) donated by Met, His, Ser, and Gly (Oommen et al., 2005; Kouzarides, 2007). Acetylation leads to the detachment of histones from DNA to favor its exposure-promoting transcription process. However, methylation plays a role in the reverse direction by densely packing the DNA and encouraging gene silencing (Wu, 2010). Studies also demonstrate the role of Gln in the regulation of intestinal gene expression in rats, promoting intestinal health concerning cell growth and antioxidation activity (Wang et al., 2008). Arg supplementation in rats leads to the upregulation of gene expression, preventing oxidative stress and promoting fatty acid metabolism and glucose metabolism (McKnight et al., 2010). At the transcriptional level, amino acids regulate the activity of RNA polymerase by altering its specificity for promoters and enhancing the binding of some repressors near the non-coding sequences adjacent to the promoter region (Oommen et al., 2005). Such studies demonstrate the remarkable contribution of different amino acids in regulating gene expression.

The human immune system consists of both innate and acquired immune subsystems that regulate the response and protection of the human body upon pathogen attack (Calder, 1995). The innate immune system is a natural system that immediately activates when pathogens enter the body and can only prevent the entry and initial establishment of the pathogen. It comprises the physiological barriers, monocytes, macrophages, neutrophils, basophils, natural killer cells, mast cells, platelets, and various humoral factors (Buchanan et al., 2006). However, once the pathogen invades the innate immune system and colonizes, the acquired immune system is activated to decrease further pathogen progress. The acquired immune system consists of lymphocytes (T- and B-lymphocytes) that have immunological memory for invading pathogens (Calder, 2006). Human immune systems require a range of amino acids to produce immunoglobulins, cytokines, and other biomolecules to prevent diseases (Kim et al., 2007).

Several amino acids (branched-chain amino acids: BCAA (Leu, Ile, and Val), alanine (Ala), Gln, Ser, Pro, and Thr) regulate the proliferation of lymphocytes (Li et al., 2007). These amino acids either directly participate (Ala, Ser, and Thr) or produce signal molecules or hormones (BCAA, Gln, and Pro) to stimulate lymphocyte proliferation and create various immune responses (Li et al., 2007). Moreover, BCAAs participate in lipid metabolism (Nishimura et al., 2010) and blood glucose maintenance. In females, BCAAs also regulate blastocyst development and embryo implantation, fetal growth by hormonal secretions, stimulate mammary gland function and lactation, and increase aspartate, Gln, and glutamate synthesis (Zhang et al., 2018). Met, His, Gly, and Phe regulate the synthesis of signaling molecules controlling immune responses. Individually or in combination, these amino acids control the production of immune cell signaling molecules, leading to major immunity-boosting elements such as cytokines and antibodies (Li et al., 2007). Amino acid oxidases (AAOs) derived from L-isomers of Phe, Trp, Tyr, and Leu possess antimicrobial (Phua et al., 2012) and antitumoral functions (Lee et al., 2014).

Legumes have antinutritional compounds, including trypsin and chymotrypsin inhibitors, phytic acids, and tannins, which reduce nutrient bioavailability (Vidal-Valverde et al., 1994; Shi et al., 2017). Lentil is naturally low in phytic acid (Thavarajah et al., 2009) and contains trypsin inhibitors (3.6–7.6 units/mg protein) and tannins (1.28–3.9 mg/g; Hefnawy, 2011). Inactivity of trypsin and chymotrypsin enzymes causes difficulties in lysis proteins into small peptides and eventually affects the release of amino acids from small peptides. Tannins are phenolic inhibitors that bind to proteins *via* Lys or Met cross-links (Davis, 1981) and make insoluble complexes with carbohydrates (Reddy et al., 1985). In lentils, trypsin and chymotrypsin inhibitors and phytic acids are present in seed cotyledons, whereas tannins are concentrated mainly in the seed coat (Dueñas et al., 2002). Different food processing methods, including dehulling and cooking, are recommended to reduce these antinutritional properties (Acquah et al., 2021). Dehulling effectively reduces the tannins by removing the seed coat (Goyal et al., 2009). In pulses, other common processing treatments are soaking, hydrothermal treatments (cooking and roasting), fermentation, and irradiation (Acquah et al., 2021). Soaking reduces trypsin and chymotrypsin inhibitors, phytic acids, and tannins in lentils depending on the soaking time (Shi et al., 2017). Thermal methods are recommended for denaturing trypsin and chymotrypsin inhibitors and removing tannin in lentils (Hefnawy, 2011). Fermentation and irradiation are alternate methods to reduce antinutritional compounds (Siddhuraju et al., 2002; Maleki and Razavi, 2021) but have not been widely studied in pulses.

## Breeding Approaches for Protein Quality Improvement

Pulse breeding programs focus on meeting the world’s food demand and ensuring global food security. The primary objectives of these breeding programs are to increase the yield by efficient selection from available germplasm, introduce hybrid lines, cross contrasting lines to exploit heterosis, develop biotic and abiotic stress-tolerant cultivars, and induce mutations to generate novel variability with molecular and genomic techniques. Today, most conventional pulse breeding programs employ molecular markers for traits of interest. Genetic engineering technology has demonstrated remarkable potential to modify plants for specific breeding objectives. Thereby, technological advancement has broadened the scope of plant breeding to enable special-purpose breeding programs such as nutritional quality improvement programs or nutritional breeding (Kumar et al., 2020).

Conventional breeding approaches focus on improving highly heritable traits governed by a few genes. Quantitative traits with low heritability and high environmental effects, such as protein and other nutritional quality traits, do not significantly respond to selection by conventional breeding methods. In crop plants, including pulses, protein concentration negatively correlates with yield (Qureshi et al., 2013); therefore, selecting either trait negatively affects the other. For this reason, conventional approaches, such as mass selection, pedigree method, and bulk method, face challenges for protein quality improvement, but adding genetic markers into the breeding pipeline is possible. A comprehensive study comparing relative protein concentration among different lentil species identified a high protein accession, ILWL 47, belonging to *L. ervoides* (Bhatty, 1986). Lentil cultivar., IC317520, was identified as a high protein, sugar, and starch cultivar (Tripathi et al., 2019). The identified candidates can improve protein content in cultivated lentils by hybridization-based breeding methods.

Compared to selection and hybridization-based methods, mutation breeding has improved legume protein. A mutant lentil variety, NIA-MASOOR-5, with increased protein concentration, high yield, and disease resistance was created by gamma irradiation of M-85 as a parent and released in Pakistan (Ali and Shaikh, 2007). Mutation using gamma radiation has increased protein levels in mutants obtained from Chiang Mai 60, SSRSN35-19-4, and EHP 275 cultivars of soybean (Yathaputanon et al., 2009). Some high-protein and low-fiber mutants were identified from gamma ray-irradiated and ethyl methanesulfonate (EMS)-treated Himso 1563 and TS 82 cultivars in soybean (Kavithamani et al., 2010). EMS also induced beneficial mutations for protein and oil content improvement in Huayu 22 and Yueyou 45 cultivars of peanut (Chen et al., 2020). A high-yielding and high-protein chickpea mutant variety, Hyprosola or Faridpur-1, was also developed by gamma irradiation in Bangladesh (Oram et al., 1987). TAEK-SAGEL is another gamma radiation-derived, high-protein mutant variety of chickpea released in Turkey (Saǧel et al., 2009). Such landmark achievements of mutation breeding in pulse crops, including lentils on a commercial scale, demonstrate the success of this method for improving quality traits.

Genomic-assisted breeding demonstrates the broad potential for improving quantitative traits, which are highly complex, controlled by many genes, and environmentally influenced (Kumar et al., 2016a). The current genomic toolbox for breeding includes genetic marker development, linkage map construction, identifying QTL and alien introgressions, candidate gene discovery, diversity analysis, genome sequencing, and pangenome construction. The use of molecular markers to gear up genomic developments in lentils for various traits has been reviewed widely (Kumar et al., 2015). Several legume crops, including dry pea (*Pisum sativum* L.), soybean, and chickpea, have been broadly investigated for use in genomic-assisted breeding to identify putative genomic regions governing seed protein concentration. The QTL mapping approach in dry pea revealed three genes regulating protein concentration using a linkage map of 207 markers (AFLP, RAPD, and STS markers; Tar’an et al., 2004). Another similar mapping study in dry pea using 204 markers (morphological, isozyme, AFLP, ISSR, STS, CAPS, and RAPD) identified genomic regions for seed protein concentration (Irzykowska and Wolko, 2004). Several other studies using genomic-assisted breeding in dry pea identified protein concentration-related genes (Tayeh et al., 2015). However, these studies are limited in the number of dry pea accessions used in each study and the genome-wide comparisons. Furthermore, a restriction-site associated DNA sequencing (RAD-seq) approach identified 47,472 SNP markers in a soybean RIL population (Liu et al., 2017), and several genes for the seed protein in soybean were found using transcriptome analysis, QTL mapping, and the genome-wide association study (GWAS) approach (Patil et al., 2017). A gene controlling seed size, weight, and composition of amino acids in total protein concentration were characterized in model legume Medicago trunculata and soybean using PCR-based markers and transcriptome profiling (Ge et al., 2016). Likewise, extensive studies in soybean have also identified several seed protein genes by exploiting genomic breeding approaches (Brummer et al., 1997; Sebolt et al., 2000; Chapman et al., 2003; Chung et al., 2003; Liang et al., 2010; Van and Mchale, 2017; Li et al., 2018; Huang et al., 2020). A high-throughput genotyping technology study identified 16,376 SNPs and revealed seven major genes for seed protein through a GWAS in 336 *desi* and *Kabuli* chickpea accessions (Upadhyaya et al., 2016). Such studies in legume crops demonstrate the success of marker-based genomic tools for improving protein concentration and quality. However, marker-based genomic-assisted studies identifying genic regions associated with seed protein content and quality have not been reported in lentils so far.

Genetic engineering technology has provided other insights to improve protein concentration in legumes. Protocols have been designed to develop transgenic lines in chickpea (Fontana et al., 1993), common bean (Russell et al., 1993), lupin (Molvig et al., 1997), peanuts (Brar et al., 1994), pea (Schroeder et al., 1993) and soybean (Hinchee et al., 1988). Several research groups have developed transgenic soybean lines with increased S-containing amino acids (Falco et al., 1995; Dinkins et al., 2001; Guo et al., 2020). Likewise, transformation studies to improve seed protein concentration in broad bean (Montamat et al., 1999), dry pea (Tegeder et al., 2007), and French bean (Tan et al., 2008) have also been reported. Recently, the genome-editing tool CRISPR/Cas 9 has emerged as a revolutionary approach to improving staple food crops, but this approach is not widespread in pulses except in soybean.

## Closing Remarks

**Figure 2:**
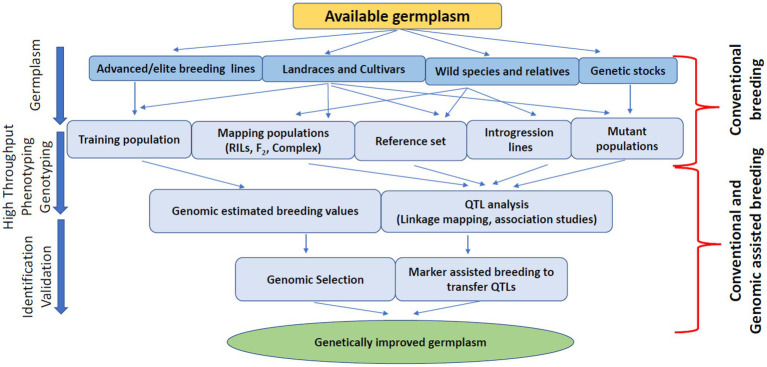
Schematic representation of germplasm improvement for quality traits.

**Figure 3:**
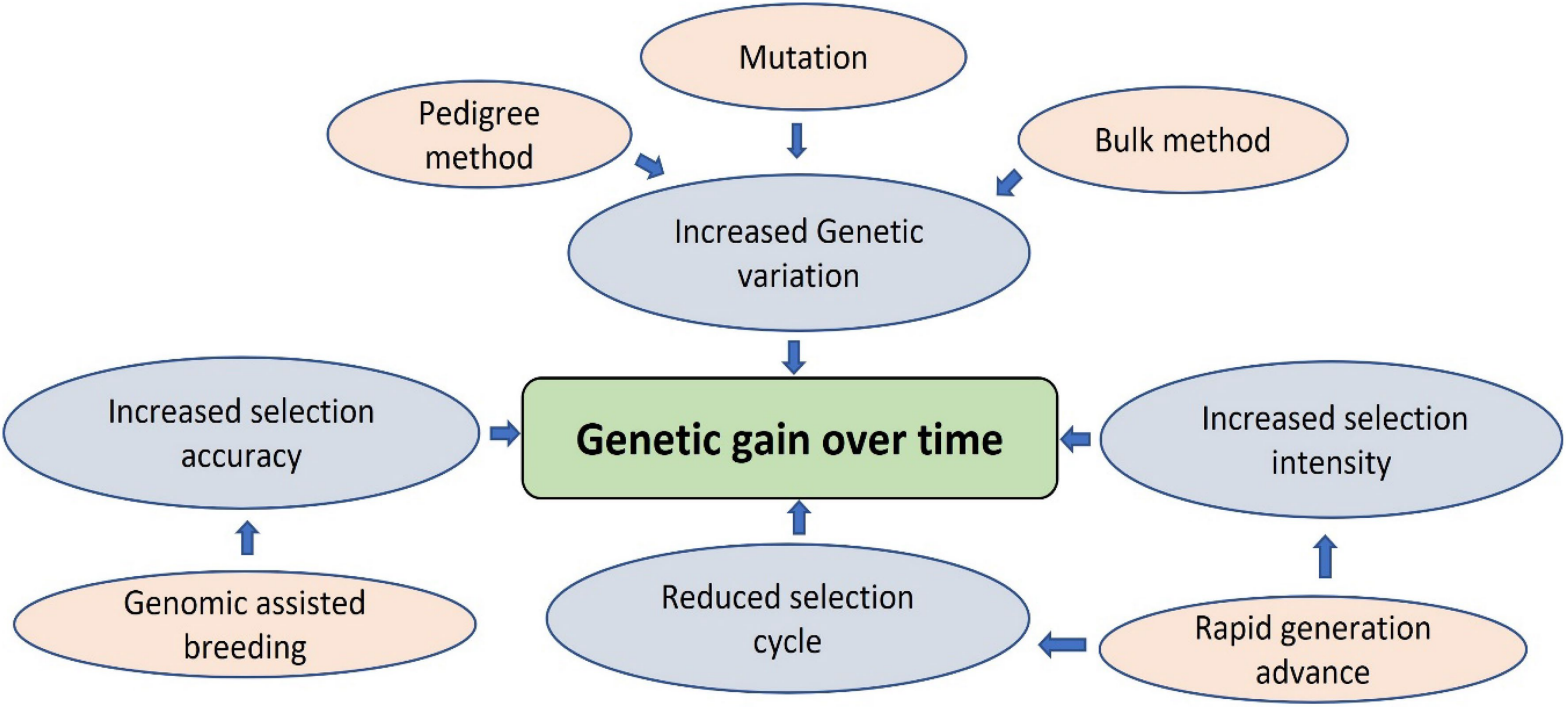
Strategies to increase genetic gain over time.

Most lentil breeding programs worldwide focus on yield improvement, disease resistance, biotic/abiotic stress tolerance, and germplasm diversity. Lentils are a nutrient-dense superfood to combat malnutrition and non-communicable diseases. As such, lentil protein quality has recently emerged as a target trait for lentil breeding programs due to the increased demand for plant-based protein. Conventional breeding is progressing for lentil crop nutritional improvement, but other genomic approaches are essential to speed up the breeding process due to the quantitative nature of these traits. Genome-wide association studies with conventional plant breeding approaches are appropriate for improving the genetic gain of quantitative traits by increasing selection accuracy through indirect selection (Rutkoski, 2019). For example, the genetic gain for lentil protein concentration can be achieved by selecting diverse parents, increasing the selection intensity, accuracy and reducing the selection cycle duration by increasing the number of generations per year. Conventional methods like pedigree, bulk, and mutation breeding can develop new breeding material using wild species, cultivars, landraces, advanced/elite breeding lines, and genetic stocks (Figure 2). These breeding methods will generate broadly diversified germplasm used for phenotyping and genotyping platforms to enhance selection accuracy (Xu et al., 2017). However, these conventional methods do not increase the selection intensity due to low heritability, slow progression, and visual phenotypic selection (Cobb et al., 2019). Combining genomic-assisted breeding with rapid generation methods such as single-seed descent, speed breeding, and double haploid production will enhance selection intensity and shorten the selection cycle, resulting in increased genetic gain over time (Cobb et al., 2019; Figure 3). Future lentil breeding efforts should focus on the rapid diversification and evaluation of lentil germplasm for protein quality through conventional breeding approaches. The development and adoption of genomic resources and tools such as genetic engineering or genome editing may also contribute to the pace of conventional breeding in lentils and eventually lead to breakthroughs in lentil protein improvement programs to ensure nutritional security and improve human health.

## Author Contributions

SS is a doctoral student under the supervision of DT who drafted the paper objectives, wrote the first draft, revised and edited the final version of this paper. JLB, PT, and SK edited/reviewed the final version and provided revisions and edits constructively. DT supervised SS and designed the objectives with SS, wrote parts of the paper, edited and revised the last version. All authors contributed to the article and approved the submitted version.

## Funding

Funding support for this project was provided by the Organic Agriculture Research and Extension Initiative (OREI; award no. 2018-51300-28431/proposal no. 2018-02799; and award no. 2021-51300-34805/proposal no. 2021-02927) of the United States Department of Agriculture, National Institute of Food and Agriculture (DT, LB), and the USDA National Institute of Food and Agriculture, [Hatch] project [1022664] (DT); the Good Food Institute (DT); and the FoodShot Global. Its contents are solely the responsibility of the authors and do not necessarily represent the official views of the USDA.

## Conflict of Interest

The authors declare that the research was conducted in the absence of any commercial or financial relationships that could be construed as a potential conflict of interest.

## Publisher’s Note

All claims expressed in this article are solely those of the authors and do not necessarily represent those of their affiliated organizations, or those of the publisher, the editors and the reviewers. Any product that may be evaluated in this article, or claim that may be made by its manufacturer, is not guaranteed or endorsed by the publisher.
